# Global cervical cancer research: A scientometric density equalizing mapping and socioeconomic analysis

**DOI:** 10.1371/journal.pone.0261503

**Published:** 2022-01-06

**Authors:** Dörthe Brüggmann, Kathrin Quinkert-Schmolke, Jenny M. Jaque, David Quarcoo, Michael K. Bohlmann, Doris Klingelhöfer, David A. Groneberg

**Affiliations:** 1 Department of Obstetrics and Gynecology and Division of Female Health and Preventive Medicine, Institute of Occupational Medicine, Social Medicine and Environmental Medicine, Goethe-University, Frankfurt, Germany; 2 Department of Obstetrics and Gynecology, Keck School of Medicine of USC, Los Angeles, California, United States of America; 3 Department of Obstetrics and Gynecology, St. Elisabeth Hospital, Loerrach, Germany; University of Central Florida, UNITED STATES

## Abstract

Cervical cancer has caused substantial morbidity and mortality for millions of women over the past decades. While enormous progress has been made in diagnosis, prevention and therapy, the disease is still fatal for many women—especially in low-income countries. Since no detailed studies are available on the worldwide research landscape, we here investigated the global scientific output related to this cancer type by an established protocol. The “New Quality and Quantity Indices in Science” platform assessed all relevant cervical cancer research published in the Web of Science since 1900. A detailed analysis was conducted including country-specific research productivity, indicators for scientific quality, and relation of research activity to socioeconomic and epidemiologic figures. Visualization of data was generated by the use of density equalizing map projections. Our approach identified 22,185 articles specifically related to cervical cancer. From a global viewpoint, the United States of America was the dominating country in absolute numbers, being followed by China and Japan. By contrast, the European countries Sweden, Austria, and Norway were positioned first when the research activity was related to the population number. When the scientific productivity was related to annual cervical cancer cases, Scandinavian countries (Finland #1, Sweden #4, Norway #5, Denmark #7), the Alpine countries Austria (#2) and Switzerland (#6), and the Netherlands (#3) were leading the field. Density equalizing mapping visualized that large parts of Africa and South America were almost invisible regarding the global participation in cervical cancer research. Our data documented that worldwide cervical cancer research activity is continuously increasing but is imbalanced from a global viewpoint. Also, the study indicated that global and public health aspects should be strengthened in cervical carcinoma research in order to empower more countries to take part in international research activities.

## Introduction

As stated by recent reviews, cervical cancer is a largely preventable female malignancy that accounts for over 300,000 worldwide deaths with more than half a million women being diagnosed every year [1, 2]. Around 85% of cervical cancers cases and 90% of related deaths occur in low- and middle-income economy settings [3]. The disease has massive global and public health implications since these countries often lack formalized HPV vaccination and cervical screening [1, 4]. The efficacy of cervical cancer screening to lower the disease burden in high-income countries is demonstrated by its impact on key epidemiology data [1]. Both, cervical cancer incidence and mortality, have decreased over the past decades beginning with the implementation of prevention programs [1, 4].

Since the malignancy is highly preventable, availability and accessibility to well-trained health care professionals are associated with a low likelihood for women to fall ill, suffer and die from cervical cancer sequelae [5]. Given the fact that the related mortality is 18 times higher in low- and middle-income compared to developed countries, the geographical location where a woman lives translates into significant regional and global disparities regarding cancer detection, treatment and related morbidity and mortality [1, 6]. This circumstance is unacceptable. Hence, the WHO released an ambitious worldwide call-to-action in May 2018 aiming to eliminate cervical cancer as a public health problem [7].

The discrepancy between efficient cervical cancer prevention leading to better survival in high-income countries and dramatic mortality in low- and middle-income countries [2, 8] suggests that related past and present scientific trends need to be interpreted in a global context. However, there is no concise worldwide analysis of cervical cancer scientific efforts of the past decades, which could help guide future research and funding strategies. In this study, the global output of cervical cancer research, which was published in the Web of Science since 1900, was investigated in the context of the NewQIS (New Quality and Quantity Indices in Science) project. This technology allows objective, precise and reliable scientometric analyses and provides the visualization of global research trends by geographic cartographs. The objectives of this study encompassed (1) an in-depth assessment of the global cervical cancer research activity by using classical publication output parameters and in terms of geographical and chronological developments, (2) the visualization of international research networks and (3) the relation of national cervical cancer research activities to socio-economic figures and cervical cancer epidemiology.

## Methods

### NewQIS technology

For this study, we used the previously established NewQIS computing platform [9, 10], which was founded in 2007/2008 at the Humboldt-University Berlin Charité by a multidisciplinary and international team of scientists. Since then, about 100 peer-reviewed studies were published using this methodology [11]. Topics ranged from infectious diseases to public and global health or gynecology and obstetrics [11]. Generally, we perform our searches employing the NewQIS computing platform according to a standardized protocol. Hence, the data obtained in this particular study can be compared to formerly published studies on other biomedical entities (e.g. ovarian and endometrial cancer) that were also based on the NewQIS platform.

### Data source

As data source, we used the Web of Science (WoS core collection, Clarivate Analytics) to analyze the world scientific productivity as represented by the body of cervical cancer research articles. This database was preferred to the PubMed search engine due to its unique WoS Citation Report [12].

### Cervical cancer search algorithm

In order to specifically assess publications related to cervical cancer, a “title” search was performed for the time period of 1900 to 2015. The search term (cervical OR cervix) AND (neoplasm* OR cancer* OR carcinom*) was used. The time after 12-31-2015 was excluded to avoid incomplete data acquisition due to the cited half-life phenomenon. Typically, the citated half-life in the field of biomedical research is 6–8 years (https://jcr.clarivate.com/jcr/browse-category-list), which leads to a dramatic decrease in citations after 2015. Therefore, the period thereafter is not suitable for valid interpretation.

On the Advanced Search interface of the Web of Science Core Collection, we restricted the analysis to the category of “articles” by setting the specific filter function to “article” [10]. This approach was chosen in order to limit the analysis to original cervical cancer research and to exclude published news, reviews or other potentially non-peer-reviewed material.

### Data analysis and categorization

As stated in previous NewQIS studies, a range of different parameters was assessed. For example, we analyzed the publication year, author, country of origin, language, subject categories, and citations. The country-specific assignment of articles was based on the affiliations the authors reported in the publications and according to the present country territories. 251 “countries” we analyzed for their productivity, which included sovereign states as well as autonomous regions. Of all analyzed countries, 154 countries contributed to the identified articles on cervical cancer. Papers that were published in a collaboration between authors affiliated with different countries were counted for each affiliated country; this circumstance was considered in the collaboration analysis. Also, *modified* Hirsch-indices were calculated as proxy measures for scientific quality. The Hirsch-index was created by *Jorge Hirsch* to measure the recognition of research performance in the scientific community. Since we did not relate the index to single authors but to countries it was termed *modified* H-index (Hi). Here, a Hi of x describes the number of x papers, which authors affiliated with a specific country published and that have been cited at least x times. Also, country-specific citation rates (CR, total country-specific citations numbers per total numbers cervical cancer publications) were computed [13].

### Socio-economic and epidemiologic analysis

A novel focus of NewQIS is the introduction of socioeconomic benchmarking among countries [14]. Cervical cancer research productivity was related to each country’s economic and demographic capabilities and cervical cancer burden. We used the following parameters for the analyses: (1) population sizes of countries (numbers of inhabitants), (2) the total economic power index gross domestic product (GDP, based on the purchasing power parity) per 1000 billion current international dollar US-$ and (3) the GDP per capita. The figures were retrieved from the CIA *World Factbook* edition of 2016 [15]. The CIA world factbook publishes population data based on the estimates from the US Bureau of the Census. They define population as people, male and female, child and adult living in a given geographical area, i. e. inhabitants (https://www.cia.gov/the-world-factbook/field/population). The number of inhabitants is used as a proxy measure for the human resources, specifically for the researchers available in a particular country, who are potentially able to generate the articles on cervical cancer. This calculation aims to compare the research output of countries in relation to the number of potential researchers in a specific field so a comparison between countries with different human resources is feasible.

In order to be able to compare categories of countries, we used the following definitions provided by the World Bank. The definitions have been based on the Gross National Income per capita in 2015, were calculated using the World Bank Atlas method (https://datahelpdesk.worldbank.org/knowledgebase/articles/906519) and included *low-income economies* (per capita income < 1.045 US-$), *high-income economies* (≥ 12.736 US-$), *lower-middle-income economies* (1.046–4.125 US-$) and *upper-middle-income economies* (4.126–12.735 US-$). To calculate ratios of publication output and R&D expenditures, we used Gross domestic expenditure on R&D (GERD) provided by the UIS UNESCO Institute of Statistics: Science, technology and innovation [16]. To assess the relative cervical cancer research activity with regard to epidemiologic data, the estimated number of new cases of cervical cancer per year for each country was used, supplied by the GLOBOCAN 2020 project of the IARC/WHO [17].

### International cervical cancer networks of research

We investigated international collaborative networks of cervical cancer research based on the main affiliations the authors stated on the published articles. A circle diagram was generated for improved visualization. Here, vectors indicate established scientific collaborations between authors from different countries; their width and shade of grey quantify the number of collaborations.

### Density equalizing map projections

A key feature of the NewQIS method is the unique visualization of the computed results [18]. Therefore, density equalizing map projections (DEMP) were calculated for the different cervical carcinoma-specific parameters resulting in a variety of anamorphic maps. The algorithm used for the DEMP creation was invented by Gastner and Newman in 2004 [19]. In this process, metadata were transferred into a Microsoft® Access® database and used for all analyses. We employed the software “ArcGIS Cartogram”geoprocessing tool (https://www.arcgis.com) to build anamorphic maps. In these cartograms, the areas of the countries were resized in proportion to the selected criteria of interest (i.e. the total number country-specific articles).

## Results

### General parameters of cervical cancer research

In total, global cervical cancer research activity between 1900 and 2015 produced a number of 22,185 original articles listed in the WoS. Of those, 20,717 (93%) were published in English, 634 (less than 3%) in German, 375 in French (1.7%), 159 in Russian (less than 1%), 119 in Spanish (less than 1%), 44 in Polish (less than 1%), 42 in Portuguese (less than 1%), 25 in Italian (less than 1%), 24 in Japanese (less than 1%), 14 in Chinese (other: N = 32).

**Fig 1:**
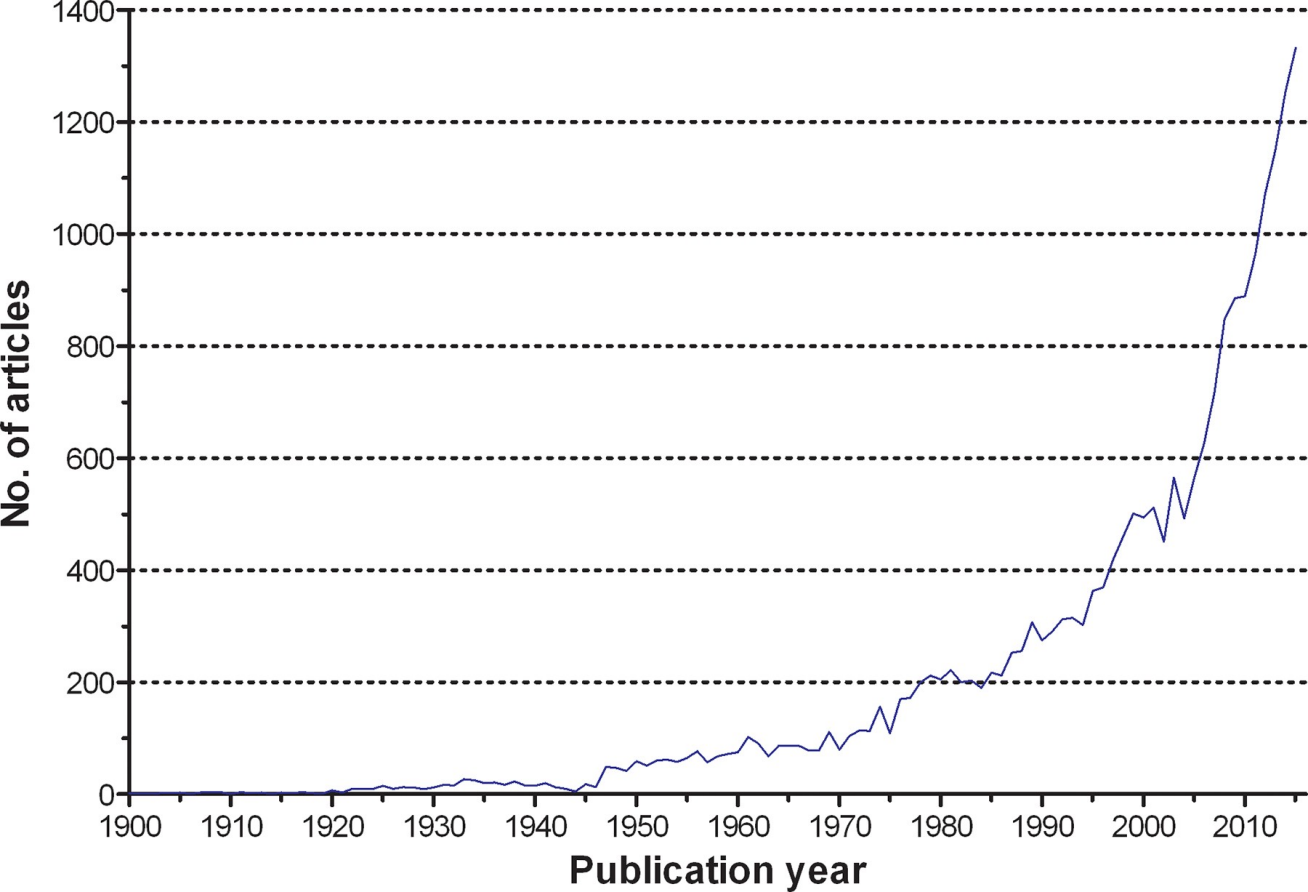
Cervical cancer research activity. Chronological development of the amount of published items per year.

Concerning the chronological development of the research activities on cervical cancer, we found only minor scientific productivity from 1900 until the 1970s. Less than 100 articles were published per year. The global research activities grew visibly in the beginning of the 1980s, when global publication activities increased to more than 200 annual articles. From 2012 on, more than 1000 papers were issued per year with a maximum number in 2015 (Fig 1).

### Cervical cancer country-specific analysis

**Fig 2:**
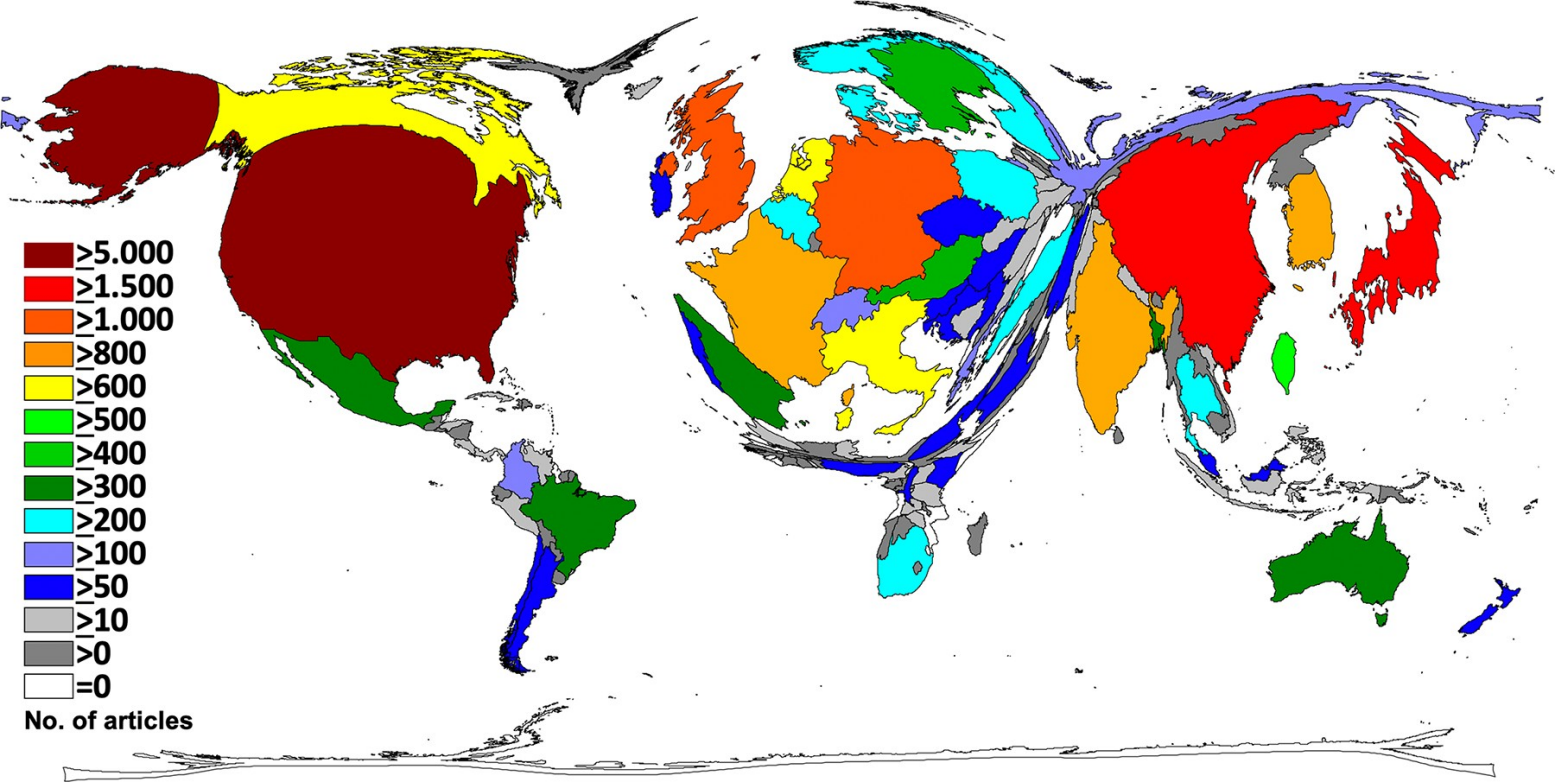
Cervical cancer research activity. Density equalizing, colours and territorial sizes indicate numbers of related articles per country.

We identified the United States of America (USA) as the country with the highest scientific productivity related to cervical cancer research; affiliated authors generated 5,640 original articles (n), which account for a quarter of all research in the investigated time period. China was ranked second (n = 1,997, 9%) and followed by Japan (n = 1,561, 7%). The most active European countries included the United Kingdom (UK; n = 1,199, 5%), Germany (n = 1,175, 5%) and France (n = 925). In total, 10 out of the 20 most active nations were *European* countries. In *South America*, Brazil was the most productive nation with 336 articles. Columbia (n = 101) and Argentina (n = 74) ranked second and third. In *Asia*, China and Japan were followed by South Korea (N = 896), India (n = 844), and Taiwan (n = 595). *Africa*´s most active countries were South Africa (n = 227), Nigeria (n = 69), and Kenia (n = 51). As visualized by DEMP cartography, we found a visible North-South divide regarding cervical cancer publication output (Fig 2).

### Cervical cancer citation analysis

**Fig 3:**
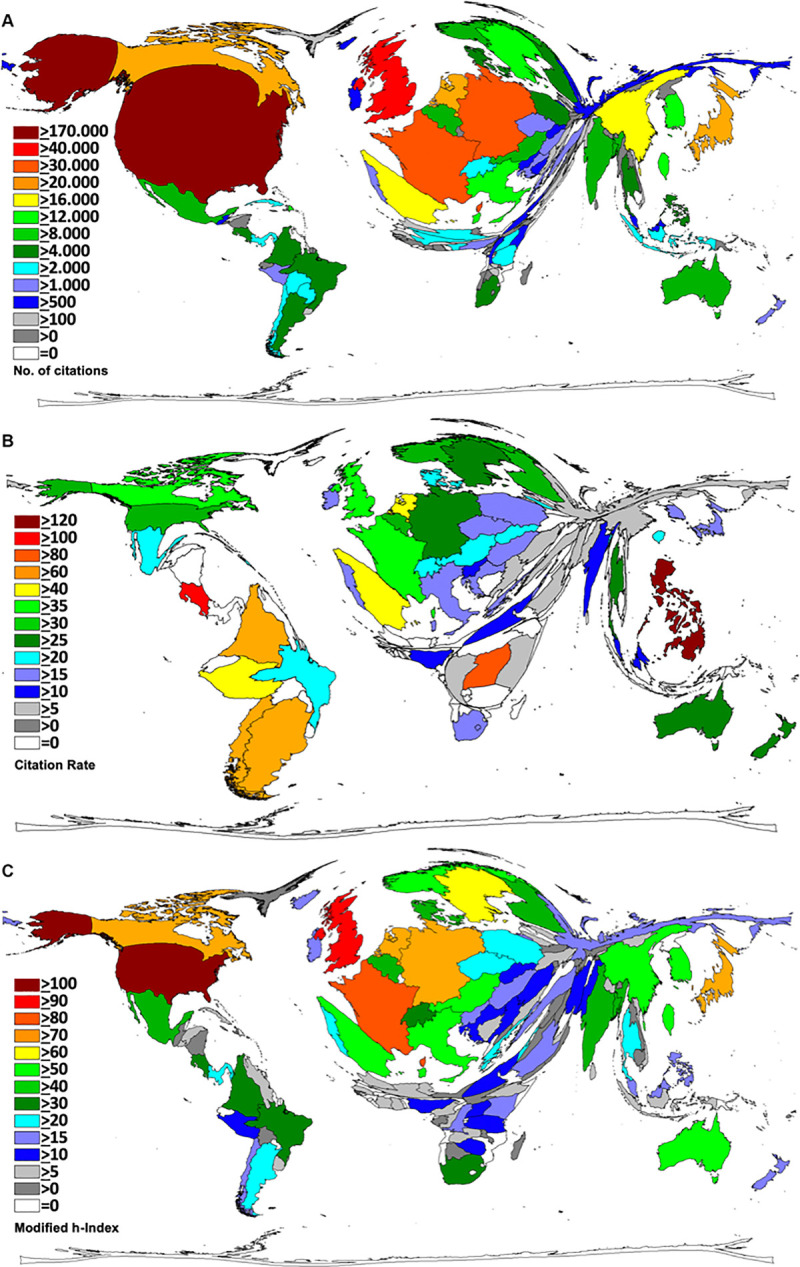
Cervical cancer research quality as represented by citation parameters. A) Total numbers of citations that articles from a respective country received. B) Density Equalizing Map of the citation rates (cr). C) Country-specific Hirsch-indices were calculated for all identified cervical cancer-specific articles.

We analyzed three citation parameters, which included the number of citations, average citation rates and the country-specific *modified* Hirsch-indices (Hi). After analysis of the metadata the results were transferred to DEMP for visualization. The **citation numbers** largely followed the pattern of the identified publication activities. The USA was leading the field. Articles published by US-American authors gained 174,754 citations (c). The *European* countries UK (c = 43.424 citations), France (c = 34.564) and Germany (c = 31.210) were ranked in second, third and fourth positions. This finding illustrates that cervical cancer studies originating from these particular countries received a higher level of recognition in the scientific community than the articles from Japan and China, which achieved 27.173 and 17.999 citations, respectively (Fig 3A).

A complete different global landscape emerged when the **average citation rates** were analyzed (threshold for countries to be included in this analysis were at least 30 cervical cancer specific articles). Here, the *East Asian* country of the Philippines was ranked first with 134.8 citations per article (cr), followed by Costa Rica (cr = 118.59) and the *African* country of Uganda (cr = 81.105). The three *South American* countries of Columbia (cr = 66.08), Chile (cr = 61.53), and Argentina (cr = 60.09) were ranked in forth, fifth and sixth position (Fig 3B). Among high-income countries, the articles of the following countries received the highest citation counts per article: Spain with a citation rate of cr = 55.35 (position 7), the Netherlands (cr = 43.07, position 9), and France (cr = 37.36, position 10). The *North American* countries Canada (position 12) and the USA (position 16) had citation rates of cr = 35.36 and cr = 30.98, respectively.

As third citation parameter, the **country-specific *modified* Hirsch-indices (Hi)** were calculated for the present set of 22,185 cervical cancer-specific articles (Fig 3C): The USA had by far the highest (Hi = 146). It was followed by the UK (Hi = 95), and France (Hi = 87). Seven out of the 10 leading nations were *Western European* counties and included the UK (Hi = 95), France (Hi = 87), Germany (Hi = 79), Sweden (HI = 63), the Netherlands (Hi = 75), Italy (Hi = 57) and Austria (Hi = 55). All of them reached a Hi above 50. Canada was ranked in 6^th^ position (Hi = 74). As representatives for the *Asian* continent, Japan (Hi = 70) was ranked 7^th^ followed by China (Hi = 53) ranked 13th. We identified South Africa (Hi = 30) as the *African* country with the highest modified Hi while Brazil (Hi = 31) was the leading country in *South America*. By comparison, *Eastern European* countries such as Slovenia (Hi = 11), Serbia (Hi = 10), Bulgaria (Hi = 7), Ukraine (Hi = 6) and Bosnia-Herzegovina (Hi = 5) were in the lower midfield of all analyzed countries. In total, we found a Hi greater than 20 for 35 countries and further 24 countries had a Hi greater or equal 10.

### Cervical cancer socio-economic analysis

To add more interesting facets to our analysis, we related cervical cancer research activity to epidemiologic and socio-economic figures in order to assess the relative contribution of countries with regard to their socio-economic and demographic power as well as their burden of disease.

We assessed the correlation between the country-specific number of research articles and the **population** represented by the number of inhabitants, which is a proxy measure for the human resources available in a particular country (Q1 = article number (n) / number of inhabitants in millions). The following three countries were ranked closely together: Sweden with 49.70 articles per inhabitants in millions (Q1), Austria (Q1 = 47.07), and Norway (Q1 = 48.29). 9 out of 10 leading countries were *European* nations with only Canada (Q1 = 25.48) in 10^th^ position being non-European. Only three *Asian* countries—South Korea (Q1 = 17.60) and Singapore (Q1 = 13.49) and Japan (Q1 = 12.32)—were among the 20 highest ranked nations. The USA published 17.41 articles per inhabitants in millions. China had a Q1 of only 1.45. As a result, the focus of leading nations shifted towards the *North/Western European* countries and particularly the Scandinavian countries (Table 1).

**Table 1:** Socioeconomic analyses.

Country	Articles	Popula-tion in mill.	Articles/Population in mill.	Rank 1	GDP in 1000 bn US-Dollar	Articles/GDP in 1000 bn US-Dollar	Rank 2
**Sweden**	491	9.88	49.70	HI 1	0.47	1044.68	HI 2
**Norway**	254	5.26	48.29	HI 2	0.36	705.56	HI 8
**Austria**	410	8.71	47.07	HI 3	0.4	1025.00	HI 3
**Denmark**	251	5.59	44.90	HI 4	0.26	965.38	HI 4
**Netherlands**	673	17.02	39.54	HI 5	0.84	801.19	HI 6
**Finland**	211	5.5	38.36	HI 6	0.22	959.09	HI 5
**Slovenia**	63	1.98	31.82	HI 7	0.06	1050.00	HI 1
**Belgium**	257	11.41	22.52	HI 8	0.49	524.49	HI 10
**Switzerland**	174	8.18	21.27	HI 9	0.48	362.50	HI 16
**Canada**	674	35.36	19.06	HI 10	1.63	413.50	HI 14
**UK**	1199	64.43	18.61	HI 11	2.7	444.07	HI 13
**South Korea**	896	50.92	17.60	HI 12	1.85	484.32	HI 11
**USA**	5640	324	17.41	HI 13	18.04	312.64	HI 21
**Australia**	398	22.99	17.31	HI 14	1.14	349.12	HI 17
**Croatia**	69	4.31	16.01	HI 15	0.09	766.67	HI 7
**Germany**	1175	80.72	14.56	HI 16	3.86	304.40	HI 22
**New Zealand**	63	4.47	14.09	HI 17	0.17	370.59	HI 15
**France**	925	66.84	13.84	HI 18	2.67	346.44	HI 18
**Singapore**	78	5.78	13.49	HI 19	0.47	165.96	HI 31
**Costa Rica**	61	4.87	12.53	UMI 1	0.07	871.43	UMI 1
**Japan**	1561	126.7	12.32	HI 20	4.84	322.52	HI 20
**Italy**	753	62.01	12.14	HI 21	3.17	237.54	HI 27
**Greece**	125	10.77	11.61	HI 22	0.28	446.43	HI 12
**Israel**	93	8.17	11.38	HI 23	0.28	332.14	HI 19
**Ireland**	52	4.95	10.51	HI 24	0.3	173.33	HI 30
**Czech Republic**	88	10.64	8.27	HI 25	0.34	258.82	HI 25
**Portugal**	88	10.83	8.13	HI 26	0.29	303.45	HI 23
**Serbia**	56	7.14	7.84	HI 27	0.1	560.00	HI 9
**Spain**	348	48.56	7.17	HI 28	1.62	214.81	HI 29
**Hungary**	70	9.87	7.09	HI 29	0.26	269.23	HI 24
**Poland**	248	38.52	6.44	HI 30	1.01	245.54	HI 26
**Slovakia**	35	5.44	6.43	HI 31	0.16	218.75	HI 28
**South Africa**	227	54.3	4.18	UMI 2	0.72	315.28	UMI 2
**Thailand**	244	68.2	3.58	UMI 3	1.11	219.82	UMI 3
**Chile**	56	17.65	3.17	HI 32	0.42	133.33	HI 32
**Mexico**	388	123.17	3.15	UMI 4	2.23	173.99	UMI 4
**Malaysia**	89	30.95	2.88	UMI 5	0.82	108.54	UMI 8
**Turkey**	219	80.27	2.73	UMI 6	1.6	136.88	UMI 6
**Saudi Arabia**	65	28.16	2.31	HI 33	1.69	38.46	HI 34
**Colombia**	101	47.22	2.14	UMI 7	0.67	150.75	UMI 5
**Romania**	45	21.6	2.08	UMI 8	0.41	109.76	UMI 7
**Argentina**	74	43.89	1.69	HI 34	0.88	84.09	HI 33
**Brazil**	336	205.82	1.63	UMI 9	3.20	105.00	UMI 9
**China**	1997	1373.54	1.45	UMI 10	19.7	101.37	UMI 10
**Kenya**	52	46.79	1.11	LMI 1	0.14	371.43	LMI 1
**Russia**	158	142.35	1.11	UMI 11	3.72	42.47	UMI 13
**Peru**	31	30.74	1.01	UMI 12	0.39	79.49	UMI 11
**Uganda**	38	38.32	0.99	LI 1	0.08	475.00	LI 1
**Iran**	81	82.8	0.98	UMI 13	1.38	58.70	UMI 12
**India**	844	1266.88	0.67	LMI 2	8.00	105.50	LMI 2
**Egypt**	51	94.67	0.54	LMI 3	1.05	48.57	LMI 4
**Nigeria**	69	186.05	0.37	LMI 4	1.09	63.30	LMI 3
**Philippines**	30	102.62	0.29	LMI 5	0.74	40.54	LMI 5
**Pakistan**	34	202	0.17	LMI 6	0.93	36.56	LMI 6

Number of cervical cancer-related articles was related to the population of each country measured in million inhabitants as well as the GDP (gross domestic product) measured in 1000 bn US-Dollar. Countries were classified in HI, LI, UMI, LM (high-, low-, upper-middle- and lower-middle-income economy countries) and ranked for each economic group. Column “Rank 1” displays the ranking for the indicator “articles per population in million”. In column “Rank 2”, results for “articles per GDP in bn US-Dollar” are ranked. The corresponding DEMP shows a distortion of the global map architecture with an inflation of regions other than in the previous DEMPs (Table 1).

In a next step, cervical cancer research was related to the **gross domestic product** (GDP) in 1000 billion (bn) US-$ (quotient Q2, Table 1). This benchmarking indicator considers the total economic power of the country and therefore gauges possible national spending in the research sector. In this ranking, Slovenia (Q2 = 1050.00) was in leading position, followed by Sweden (Q2 = 1044.88) and Austria (Q2 = 1025). One out of the first six countries did not belong to the category of high-income countries (Costa Rica, Q2 = 871.43). Denmark (Q2 = 1267.14) and Finland (Q2 = 1207,14) were listed in position 4 and 5. In total, 11 different countries had a calculated Q2 of greater or equal 500 cervical cancer articles per GDP (in 1000 bn US-$). Out of these, 3 countries were located on the *Eastern European* and 1 on the *South American* continent (Table 1).

We also assessed the ratio of number of articles to the **GDP per capita**, which should represent the number of articles in relation to the economic strength of every individual. Here, China (Q3 = 154.8) and India (Q3 = 145.5) as upper-middle- (UMI), and lower-middle-income economy (LMI) countries were ranked before the USA. With China, India, Japan (Q3 = 41.2) and South Korea (Q3 = 25.3), four countries out of the top ten were Asian countries. This leading role of countries located on the *Asian* continent was a remarkable finding. To capture the ratio of publications output to research and development activities, the indicator Gross Expenditure on Research and Development (GERD) was used (Table 2). In this approach Uganda, Cost Rica, Peru, Serbia and Columbia were listed in the top five.

**Table 2:** Ranking of the number of articles per Gross Expenditure on Research and Development (GERD) of publishing countries with at least 30 articles (threshold).

Country	Articles	GERD in 1000 current PPP$	GERD in billion current PPP$	Articles/GERD in billion current PPP$	Rank 3
**Uganda** **	38	109960,6816	0,11	345,58	LI 1
**Costa Rica**	61	361778,6579	0,36	168,61	UMI 1
**Croatia**	69	812679,4379	0,81	84,90	HI 1
**Peru**	31	412634,0066	0,41	75,13	UMI 2
**Serbia**	56	859158,7798	0,86	65,18	HI 2
**Colombia**	101	1826591,212	1,83	55,29	UMI 3
**Greece**	125	2798161,296	2,80	44,67	HI 3
**Slovenia**	63	1433393,599	1,43	43,95	HI 4
**Norway**	254	6063453,917	6,06	41,89	HI 5
**South Africa**	227	5551117,362	5,55	40,89	UMI 4
**Mexico**	388	9577903,517	9,58	40,51	UMI 5
**Netherlands**	673	16913370,74	16,91	39,79	HI 6
**Thailand**	244	6698278,144	6,70	36,43	UMI 6
**Chile**	56	1552182,001	1,55	36,08	HI 7
**Sweden**	491	15493207,87	15,49	31,69	HI 8
**Finland**	211	6689667,484	6,69	31,54	HI 9
**Austria**	410	13146956,11	13,15	31,19	HI 10
**New Zealand**	63	2125427,322	2,13	29,64	HI 11
**Denmark**	251	8517954,042	8,52	29,47	HI 12
**UK**	1199	45678218,86	45,68	26,25	HI 13
**Philippines**	30	1150918,385	1,15	26,07	LMI 1
**Italy**	753	30002911,33	30,00	25,10	HI 14
**Canada**	674	27005522,42	27,01	24,96	HI 15
**Poland**	248	10234761,37	10,23	24,23	HI 16
**Portugal**	88	3820807,469	3,82	23,03	HI 17
**Romania**	45	2091472,249	2,09	21,52	UMI 7
**Belgium**	257	12651165,81	12,65	20,31	HI 18
**Hungary**	70	3534537,667	3,53	19,80	HI 19
**Australia**	398	21151512,23	21,15	18,82	HI 20
**Slovakia**	35	1886935,128	1,89	18,55	HI 21
**Iran**	81	4454361,066	4,45	18,18	UMI 8
**Spain**	348	19820573,55	19,82	17,56	HI 22
**India**	844	49624344,95	49,62	17,01	LMI 2
**Pakistan**	34	2145827,7	2,15	15,84	LMI 3
**France**	925	61645623,01	61,65	15,01	HI 23
**Argentina**	74	5399222,385	5,40	13,71	UMI 9
**Ireland**	52	3840109,998	3,84	13,54	HI 25
**Czech Republic**	88	6854793,638	6,85	12,84	HI 26
**Turkey**	219	17739024,78	17,74	12,35	UMI 10
**USA**	5640	495094000	495,09	11,39	HI 27
**Germany**	1175	114128211	114,13	10,30	HI 28
**Switzerland**	174	17854924,82	17,85	9,75	HI 29
**Malaysia**	89	9605799,041	9,61	9,27	UMI 11
**Japan**	1561	168546118,8	168,55	9,26	HI 30
**Brazil**	336	40477269,63	40,48	8,30	UMI 12
**Singapore**	78	10504174,62	10,50	7,43	HI 31
**Israel**	93	12666266,41	12,67	7,34	HI 32
**Egypt**	51	7647413,207	7,65	6,67	LMI 4
**China**	1997	366080932,2	366,08	5,46	UMI 13
**Saudi Arabia** *	65	13696027,64	13,70	4,75	HI 33
**Russia**	158	38818630,28	38,82	4,07	UMI 14
**Kenya**	52	–	–	–	–
**Nigeria**	69	–	–	–	–
**South Korea**	896	–	–	–	–

PPP$ = purchasing power parity in US-Dollars,— = no data available

* Saudi Arabia: available value from 2013

** Uganda: available value from 2014, HI = High-income economy country, UMI = Upper-middle-income economy country, LMI = low-middle-income economy country, LI = low-income economy country, sorted by Article/GERD in bn current PPP$. Source of GERD: UNESCO Institute of Statistics: Science, technology and innovation [16].

**Fig 4:**
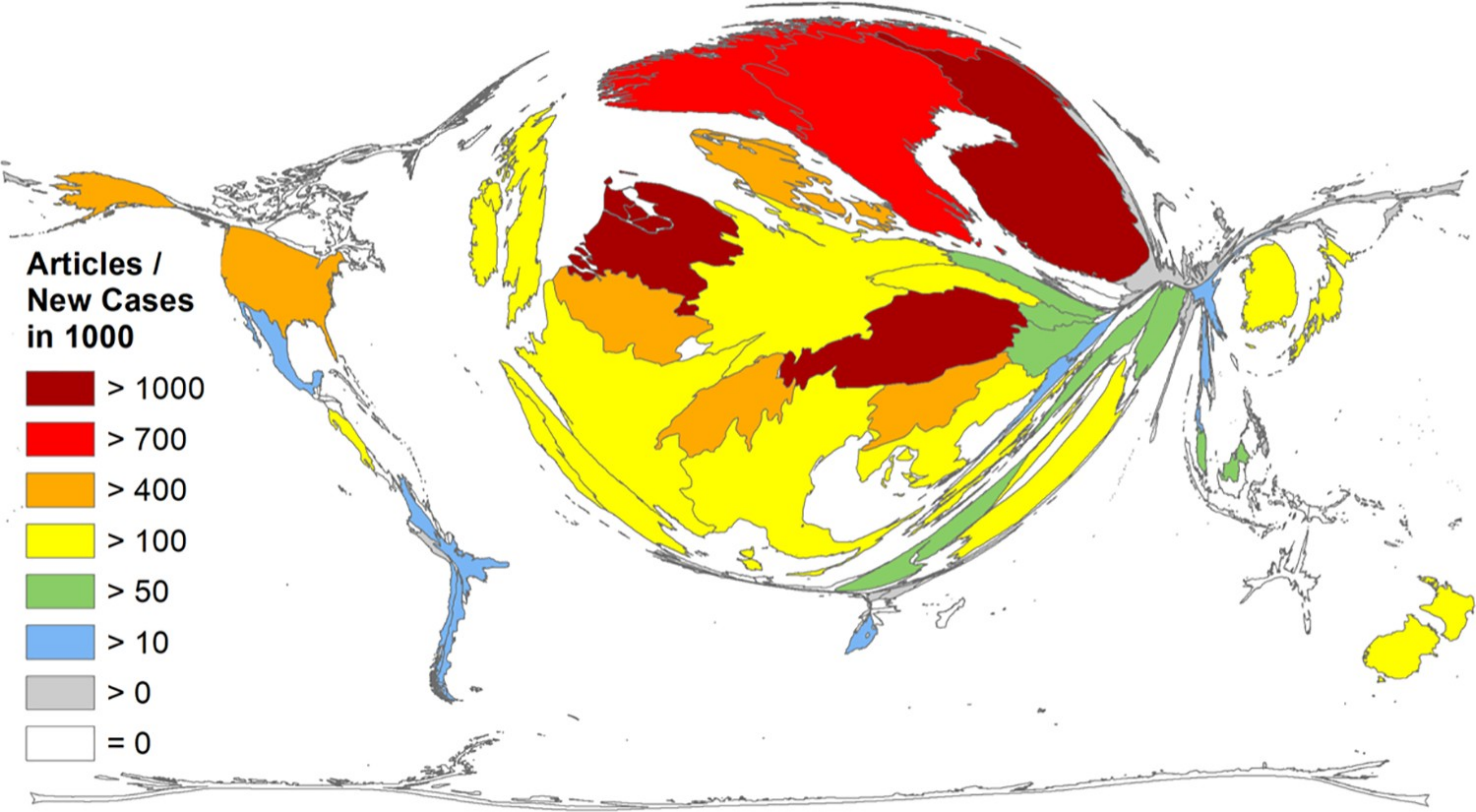
Epidemiologic analysis of cervical cancer research. DEMP of research activity measured in cervical cancer-related articles per estimated annual new cases of cervical cancer in 1000.

At last, an epidemiologic figure, we calculated the Q4 ratio relating the number of published articles to annual **new cervical cancer cases in 1000** (Table 3, Fig 4). In this ratio, Scandinavian countries (Finland #1, Sweden #4, Norway #7, Denmark #6), the Alpine countries Austria (#2) and Switzerland (#5), and the Netherlands (#3) were ranked among the seven leading countries. The leader in the field, Finland, published 1140.54 articles per annual new cervical cancer in 1000 cases whereas the USA and China were visibly less active with only 416.39 and 18.20 articles per annual new cervical cancer cases in 1000, respectively (Table 3).

**Table 3:** Epidemiologic analysis of cervical cancer research.

Country	Estimated annual new cases	Estimated annual new cases in 1000	Articles	Articles/estimated new cases in 1000 per year
**Finland**	185	0.185	211	1140.54
**Austria**	385	0.385	410	1064.94
**Netherlands**	773	0.773	673	870.63
**Sweden**	656	0.656	491	748.48
**Switzerland**	236	0.236	174	737.29
**Denmark**	384	0.384	251	653.65
**Norway**	397	0.397	254	639.80
**Slovenia**	104	0.104	63	605.77
**Canada**	1422	1.422	674	473.98
**South Korea**	1970	1.97	896	454.82
**Australia**	920	0.92	398	432.61
**USA**	13545	13.545	5640	416.39
**Belgium**	639	0.639	257	402.19
**Israel**	245	0.245	93	379.59
**New Zealand**	174	0.174	63	362.07
**UK**	3791	3.791	1199	316.28
**France**	3379	3.379	925	273.75
**Singapore**	309	0.309	78	252.43
**Germany**	4666	4.666	1175	251.82
**Italy**	3152	3.152	753	238.90
**Croatia**	336	0.336	69	205.36
**Saudi Arabia**	358	0.358	65	181.56
**Greece**	697	0.697	125	179.34
**Spain**	1957	1.957	348	177.82
**Costa Rica**	367	0.367	61	166.21
**Ireland**	342	0.342	52	152.05
**Japan**	12785	12.785	1561	122.10
**Czechia**	769	0.769	88	114.43
**Portugal**	865	0.865	88	101.73
**Turkey**	2532	2.532	219	86.49
**Iran**	1056	1.056	81	76.70
**Poland**	3862	3.862	248	64.22
**Hungary**	1251	1.251	70	55.96
**Malaysia**	1740	1.74	89	51.15
**Slovakia**	698	0.698	35	50.14
**Serbia**	1205	1.205	56	46.47
**Mexico**	9439	9.439	388	41.11
**Egypt**	1320	1.32	51	38.64
**Chile**	1503	1.503	56	37.26
**Thailand**	9158	9.158	244	26.64
**Colombia**	4742	4.742	101	21.30
**South Africa**	10702	10.702	227	21.21
**Brazil**	17743	17.743	336	18.94
**China**	109741	109.741	1997	18.20
**Argentina**	4583	4.583	74	16.15
**Romania**	3380	3.38	45	13.31
**Russia**	15308	15.308	158	10.32
**Kenya**	5236	5.236	52	9.93
**Peru**	4270	4.27	31	7.26
**India**	123907	123.907	844	6.81
**Pakistan**	5008	5.008	34	6.79
**Nigeria**	12075	12.075	69	5.71
**Uganda**	6959	6.959	38	5.46
**Philippines**	7897	7.897	30	3.80

Estimated annual number of new cases as supplied by the GLOBOCAN 2020 project of the IARC/WHO [17].

### Network analysis of international cervical cancer research

**Fig 5:**
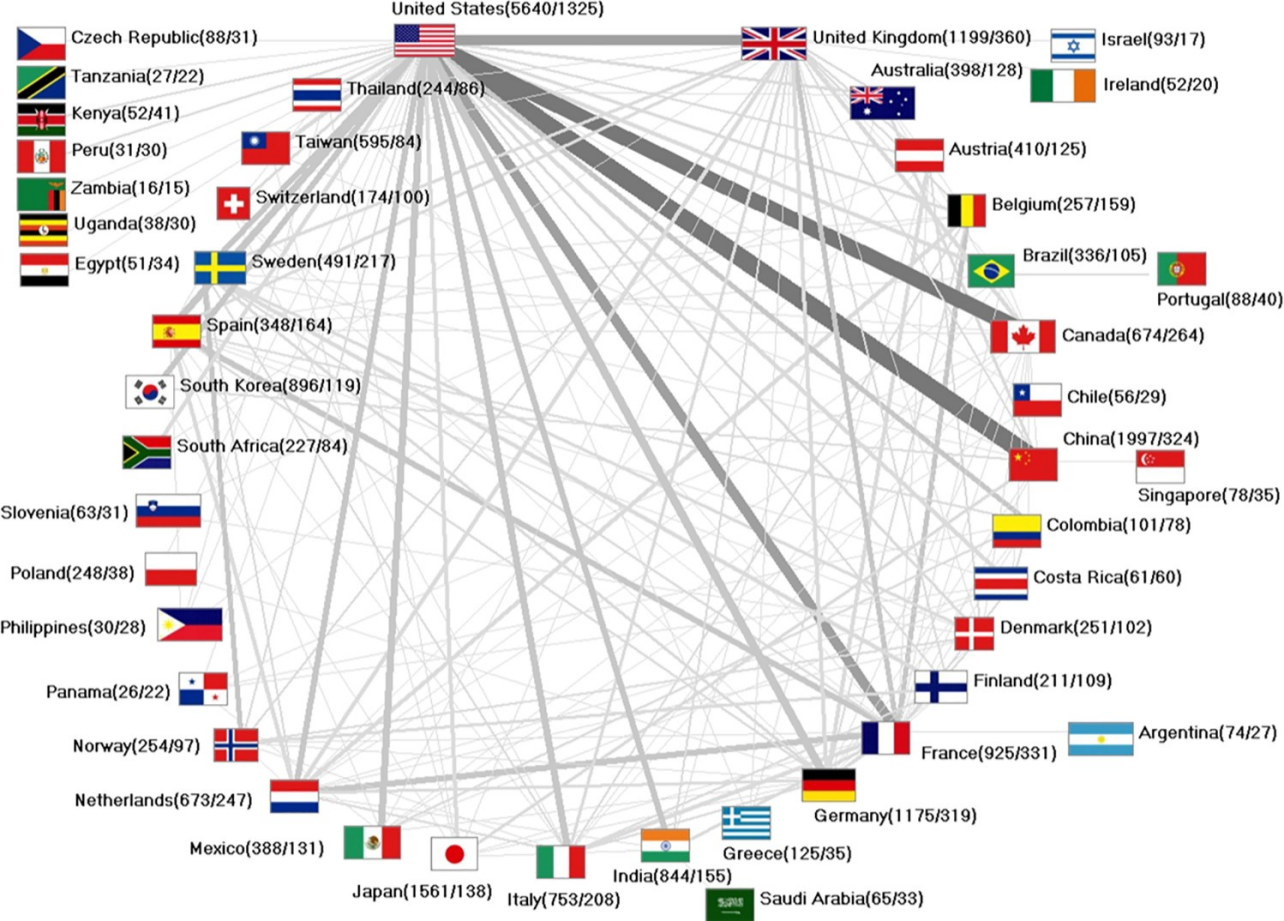
International network on cervical cancer research. Collaborating countries with ≥ 10 joint bilateral articles. Values in brackets: number of articles / number of collaborating articles. Thickness of bars corresponds to number of bilateral collaborations between linked countries.

2,069 cervical cancer articles and therefor the majority originated from bilateral scientific collaborations. Trilateral cooperative studies produced 363 publications. Collaborative efforts by four countries generated 108 articles, 70 papers were issued by authors affiliated with five countries. 1,325 collaborations involved US-American authors, followed by cooperations (number of collaborative articles = n_coop_) by scientists from the UK (n_coop_ = 360), France (n_coop_ = 331), China (n_coop_ = 324), Germany (n_coop_ = 319), and Canada (n_coop_ = 264) (Fig 5).

### Cervical cancer research area analysis

**Fig 6:**
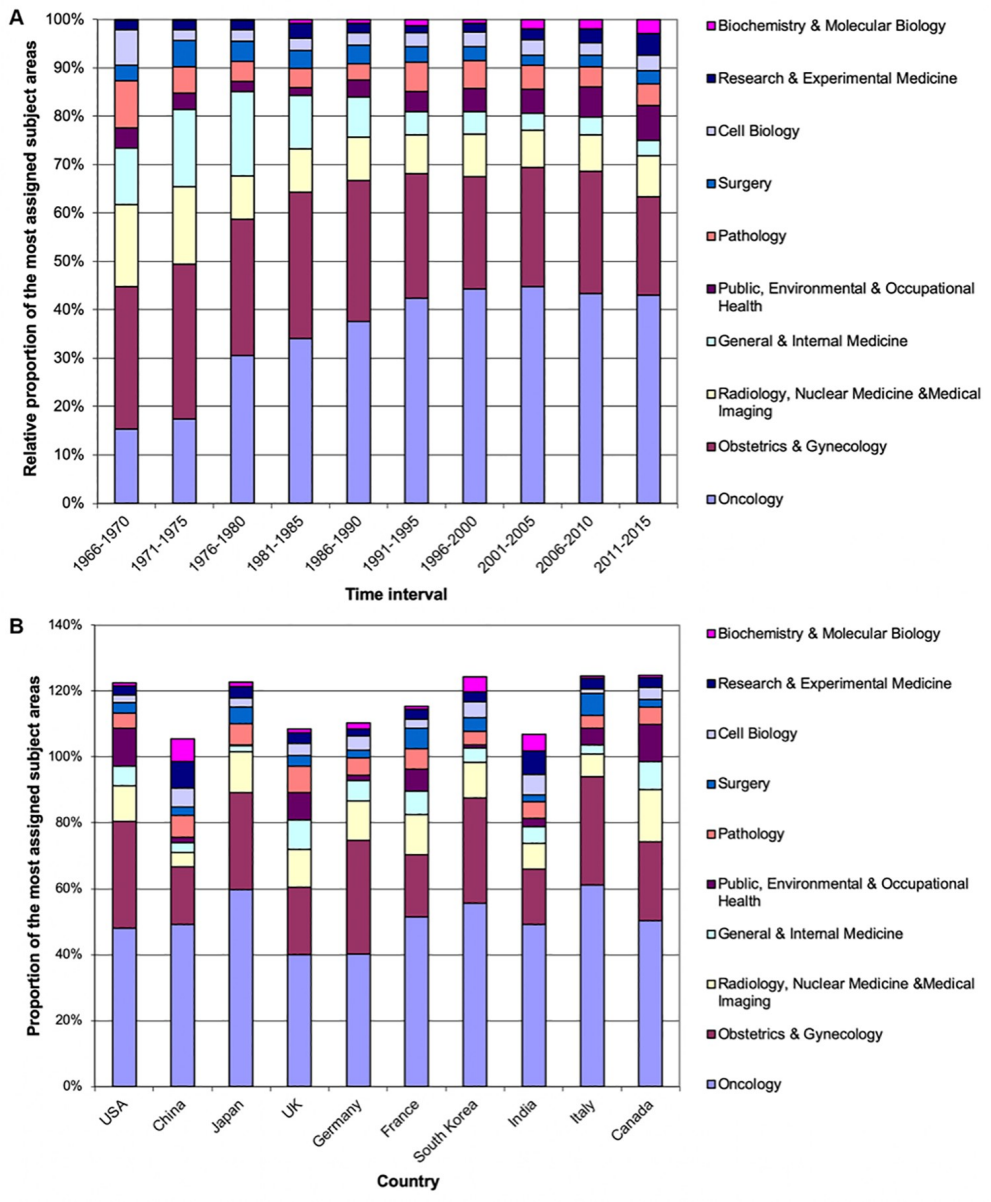
Subject areas of cervical cancer research. A) Relative proportion of the most assigned subject areas in 5-year intervals. B) Proportion of the most assigned subject areas of cervical cancer research.

Important information can be drawn from the subject category analysis of published cervical cancer research. Here, the leading field in cervical cancer research was attributed to *Oncology* (9,962 publications, n). This finding was not surprising. The second most articles were found in the subject area *Obstetrics & Gynecology* (6.579 articles). After a large gap, the ranking continued with areas such as *Radiology*, *Nuclear Medicine & Medical Imaging* (n = 2,343), *General & Internal Medicine* (n = 1,612), *Public*, *Environmental & Occupational Health* (n = 1,280), and *Pathology* (n = 1,186). The analysis of the relative share of specific subject areas in the total publication output over the time demonstrated that there was a continuous increase in the field of *Oncology* until the time period from 1996 to 2000. Since then, 40–50% of articles on cervical cancer remain attributed to this predominant area. During the period from 2011 until 2015, the field of *Obstetrics & Gynecology* was relatively unpopular in comparison to earlier periods. Among the subject areas with more than 1,000 publications, the field of *Public*, *Environmental & Occupational Health* (n = 1,280) had the strongest relative increase over the past periods (Fig 6A).

Lastly, a subject area analysis with regard to the ten most active countries was performed to deduce country-specific research interests. The fields *Oncology* followed *by Obstetrics & Gynecology* were among the most popular scientific areas. Both areas dominated with varying shares, e.g. in Norway, more than 60% of all articles were related to *Oncology* whereas in in the UK, only about 40% of articles were related to this field (Fig 6B).

## Discussion

From the viewpoint of epidemiology, cervical carcinoma can be characterized as being *“Janus-faced”*. A substantial decline of cervical carcinoma mortality and incidence was associated with newly implemented population-based screening programs in particular regions of the world such as Australia (e.g. New South Wales) [20]. On the other hand, cancer incidence and mortality rates in low-income countries without screening programs and novel therapeutic options remain high. This good and ill facet of cervical cancer demonstrates the necessity for a global approach to this topic. But how are worldwide scientific efforts distributed in this particular area of gynecologic oncology? To depict the global research landscape, this NewQIS project aimed to analyze all relevant articles in cervical cancer research. Scientometric data were related to socioeconomic figures and results were visualized using density equalizing map projections, which were established by Newman and Gastner in 2004 [19]. In order to overcome a too simplistic bibliometric approach, we calculated Hirsch-indices [13] on the basis of the citations numbers articles of each country gained and related research productivity to relevant socio-economic figures.

We identified 22,185 cervical cancer articles published between 1900 and 2015 by a specific search term that was present in the title of each identified article. In order to consider only scientific studies, the analysis was focused on original articles and excluded reviews, letters and other potentially non-peer-reviewed publications. The data acquisition was based on the Web of Science. This particular database was chosen because it includes only curated high-quality publications after a rigorous selection process ensuring a high level of scientific integrity.

Although the total output on cervical cancer continuously increased since 1900 a large imbalance of research activities was identified; most scientific activities were generated in countries located in the Northern hemisphere—specifically on the *Northern American* and the *European* continent. Nations with a high cancer burden and mortality such as Latin American, or African countries play almost no visible role among the research power players in the field and were rarely part of productive research collaborations. This observation corresponds with the extremely high mortality women face in these countries and represents a major health related social disparity. This represents a need to be addressed. Involving non-high-income countries into scientific collaborations could represent an opportunity for the transfer of ideas, knowledge or epidemiological data and therefore used to mutual advantage. Furthermore, this study underlines that particular countries, e.g. the Scandinavian nations, successfully address the public cancer burden by fostering research in the respective field. In our socioeconomic analyses, they were leading the field by publishing many research articles related to their population, GDP and cervical cancer cases–indicating an effective public health strategy.

Pivotal milestones in cervical cancer research and publication of national guidelines can spark interest and drive related scientific productivity. The identification of HPV as a causative agent for cervical cancer in 1975 [21] laid the groundwork for formalized cancer screening programs, HPV-based diagnostics and vaccines. Hence, the decades from 1980 to 2010 were characterized by the translation of novel research findings into relevant public health measures. For example, the ACOG and the American Cancer Society published the first joint consensus statement regarding formalized cervical cancer screening in 1988 [22]. In 1988 the first HPV-test was approved in the USA, after 2006 HPV vaccines were licensed in the USA and Canada for primary prevention [23]. In this study, analysis of chronological developments in the cervical cancer research output reflects these pivotal milestones: A striking increase in the number of annual publications was documented after 1975. Here, the output more than doubled to 180–300 articles on cervical cancer per year. Also, 6 of the 20 most cited articles were published in the ´80s, and in 1999 the most cited article of all times -“Human papillomavirus is a necessary cause of invasive cervical cancer worldwide”- was issued by Walboomers et al. [24]. In 2000 until 2011—the period of established HPV-based testing and vaccination—the number of published articles doubled again from 494 to nearly 1000 per year documenting a profound global interest in cervical cancer related research.

To estimate whether a number of 22,185 cervical cancer articles corresponds to a high or low activity from a global viewpoint, it was compared to published data on other gynecological cancers, such as breast [25], ovarian [26] and endometrial cancer [27]. When contrasting the present approach to the study of Glynn et al. on *breast cancer*, three large differences appear to be important: 1) breast cancer has a differing epidemiology and therefore we should expect different research activities, 2) the study of Glynn et al. covered only the years from 1945 to 2008, which is a notably shorter time period, and 3) the authors performed a broader “topic” search. Therefore, in terms of overall research activity it is not justified to compare the total number of 180,126 publications related to breast cancer to the identified 22,185 articles on cervical cancer. However, looking at chronological trends in the scientific productivity it can be stated that researchers in breast cancer demonstrated a steep increase in their output since 1945 with the USA as the leading force, followed by the UK, Germany, Italy and Japan. A similar pattern was seen in the field of cervical cancer where the global scientific output has been rising for the last 115 years (particularly since 1945) and countries such as the USA, Japan, the UK and Germany also occupy outstanding positions in the respective research community.

The previously published data on *ovarian cancer* research productivity [26] reported a number of 23,378 articles and is more similar in terms of the underlying methodology. This study covered the years from 1900 to 2014, so the period was shorter by only one year. Also, the search was restricted to articles as document types and to title words. With the difference of one year– 1332 articles related to cervical cancer were published in 2015 –we may compare the overall data: From 1900 to 2014, 20,855 cervical cancer articles represent 89.2% of the global ovarian carcinoma research activity (n = 23,378). If we relate these numbers to the estimated annual new cases of both cancer types, i.e. in the USA for the year 2014 (n = 12,722 for cervical cancer and n = 21,495 for ovarian cancer, https://gis.cdc.gov/Cancer/USCS/DataViz.html) we find a ratio of 0.59. So, nearly 1.7-times more ovarian cancer cases than cervical cancer cases occur in the USA per year. Hence, it can be deduced, that the ratio between annual cases of both cancers is not reflected by the ratio between respective scientific research productivities. This observation may lead to the assumption that scientific efforts on cervical cancer might be over-supported compared to those on ovarian cancer. In 2012, Carter and Nguyen made a statement that points in the same direction [28]. The authors concluded that cervical cancer is an overfunded condition when incidence, mortality and “Years of Life Lost" were considered. However, when the specific US-American research activity in both entities is considered (5,640 cervical cancer articles versus 9,312 ovarian cancer articles) and not the global numbers, a ratio of 60.6% can be calculated, which is very similar to the ratio of new cases. Therefore, we might come to the conclusion that US-American research activities are very much balanced towards the comparative epidemiology and public cancer burden of both female cancer types. The next question points to the direction of country-specific differences in ovarian versus cervical cancer research activities. If we compare both cancer types (Table 4) it is obvious that the research on cervical cancer is more balanced and globally more evenly distributed. In this respect, the top ten countries account for 76.62% of all articles in ovarian cancer research while they only account for 65.46% of all articles in cervical cancer research. However, focusing on the specific participation of developing countries in ovarian and cervical cancer research a similar pattern was found for both entities. Whereas strong output was generated in countries located in North America, Europe as well as in China, Japan and Australia only minor productivity was documented in countries on the South American continent and in Africa. Particularly, women in South America experience an exceptional burden due to both cancers. Besides the striking burden related to cervical cancer, South American women also face an intermediate age-adjusted incidence rate of 5,8 per 100,000 for ovarian cancer [29]. Hence, the low local research productivity and the rare involvement in international collaborations regarding both cancer types call for improvement. So South American countries could strengthen their scientific efforts for locally relevant cancers—like they already have in other biomedical fields such as toxoplasmosis research [14].

**Table 4:** Comparison of country specific research activities between ovarian cancer research (1900–2014) and cervical cancer research (1900–2015).

Ovarian cancer		Cervical cancer	
US	9,312	US	5,640
UK	1,900	China	1,997
China	1,813	Japan	1,561
Germany	1,717	UK	1,199
Japan	1,673	Germany	1,175
Italy	1,672	France	925
Canada	1,286	South Korea	896
France	878	India	844
Netherlands	762	Italy	753
Australia	742	Canada	674
**Top10**	**21,013**	**Top10**	**14,237**
Other countries	7,013	Other countries	8,266
**Total**	**28,768**	**Total**	**23,930**

Ovarian cancer data from [26]. The accumulated number of articles in this table is higher than the total number of published articles since one published article can be published by authors of more than one country.

*Endometrial cancer* is a third female carcinoma that may be compared to the presented study [27]. Within the same time period (1900–2015) and a similar search structure–identification of endometrial cancer articles by title word search—a total of 9,141 articles were found in the WoS. This corresponds to less than 50% of the identified cervical cancer research output. Among the global research power players on endometrial cancer, the USA dominated the field with more than a third of the worldwide research productivity since 1900 (n = 3,191). This equals only 56.6% of cervical cancer research issued by US-American authors (n = 5,640) during the same time period. At position 2 and 3, Japan (n = 1,074) and Chinas are listed (n = 611) for endometrial cancer, pointing to a similar pattern of the most active countries in both fields [27]. When compared to endometrial cancer, it becomes apparent that cervical cancer research is firmly established in the subject area of “Public Health”. A dramatic decrease in “Public Health” research related to endometrial cancer was documented since 1966 whereas cervical cancer articles attributed to this area achieved the strongest increase over the past decades. Thus, a solid foundation has been established, which can be used by clinicians and researchers to answer the WHO’s call-to-action aiming at the elimination of cervical cancer.

Additionally, one might also compare the present data to research on benign female neoplasms such as *uterine fibroids/myomas* [18]: This analysis covered the identical time span from 1900 to 2015; a title search was performed. However, it was not restricted to articles but included all other publications. Therefore, it is difficult to compare the data. Re-analysis showed that 4,293 of the 6,176 uterine fibroid-related publications were articles. This number might be related to the presently identified 22,185 cervical cancer-related articles and clearly indicates that global research activities on this benign disease were much lower. Lower scientific productivity was also present when socioeconomic ratios were taken into account, i.e. in the country-specific uterine myoma ranking concerning the population size, the highest activity was assessed for Finland with 12.55 publications per million inhabitants, followed by Israel (Q1 = 10.36), Sweden (Q1 = 7.92), and Belgium (Q1 = 6.90). In contrast, calculated ratios of cervical cancer article number per million inhabitants were by far higher in Sweden, Austria and Norway reaching a ratio of about 50 cervix cancer articles.

It would be reasonable to compare the global scientific productivity on *HPV research* [30] to data on cervical cancer output. But this comparison has several limitations. Firstly, the analysis of scientific activity on HPV covered a shorter period (1900 to 2009) and the search was broader because it was performed in abstract, title and keywords (“topic search”). Secondly, when country-specific research patterns on HPV were contrasted to cervical cancer, China seems under-represented in the field of HPV. This result has to be seen critically since the analysis stopped in 2009 and China has dramatically increased publication output in various fields of medicine ever since [31–33].

It is a unique strength of this study to depict the global scientific productivity on cervical cancer over a period of 115 years. Some limitations can be identified in this study: The WoS data bank focuses mainly on publications written in English and presents publications related to the search term in a curated way. Hence, we acknowledge that our search cannot identify **all** articles published on cervical cancer since 1900. Further, we must assume an underrepresentation of non-English literature in this analysis although we identified numerous articles in other languages than English. However, we deem this bias as limited since English is considered as the “language of science” and also not-native speakers publish their high-quality science in English journals. We also acknowledge that other platforms such as EMBASE or Google Scholar catalogue journal articles published in the medical field. Hence, these search engines could identify a different set of articles related to the search terminus. But these other platforms cover different time periods, show inconsistent accuracy in the citation analysis [34] and do not offer unique tools such as the Journal Citation Reports so we refrained from using them. Further, we tried to gauge scientific quality by citation parameters, which might be difficult since citations represent *recognition* of scientific activities in the research community and could be skewed by phenomena such as the Matthew effect or self-citations. Lastly, a relation of the country-specific research output since 1900 to the GDP in 2015 aimed to account for the economic capabilities to invest in research and related infrastructure. Here, it has to be acknowledged that the GDP of certain countries changed dramatically during the last decades. Since there is a positive relationship between the GDP and the scientific output in developed and developing countries [35] we consider the impact of these GDP changes over time as insignificant for the scientific value of the analysis.

## Conclusions

Our study on cervical cancer research activity documented a continuous worldwide increase in scientific productivity since 1900. The results documented a striking global asymmetry with the Southern hemisphere being underrepresented in the scientific community although the public burden of cervical cancer remains high in these countries. Scandinavian countries were identified as effective in their research endeavors since they published high article numbers related to the local cervical cancer burden and human resources. Still, many research needs remain worldwide and have to be addressed defying geopolitical, cultural and environmental challenges. Researchers will be more successful embedded in international collaborations, which need to be facilitated by funding programs. So, health disparities will be diminished when countries are empowered to improve prevention, diagnosis and treatment of cervical cancer according to their capabilities.
